# Outward investment of Portuguese small and medium enterprises in the Central and Eastern European countries: motivations and challenges

**DOI:** 10.12688/f1000research.122697.1

**Published:** 2022-11-17

**Authors:** Eleonora Santos, Jacinta Moreira

**Affiliations:** 1 Centre of Applied Research in Management and Economics, Polytechnic Institute of Leiria, Leiria, 2411-901, Portugal

**Keywords:** Outward foreign direct investment, CEECs, Sttategies, Internationalization

## Abstract

**Background:** This paper identifies the determinant factors of Portuguese investment in Poland, Hungary, and the Czech Republic. We assume that investment abroad is motivated by business opportunities, and the quality-price ratio of the workforce.

**Methods:** To this end, we used a qualitative methodology composed of 6 case studies, based on interviews and surveys with the managers of the Portuguese firms investing in those three economies.

**Results:** Despite the business opportunities, Portuguese investment directed towards these economies is negligible, due, in part, to the geographic and cultural distance. However, the economic and political stability, combined with market size and growth potential are undeniable attraction factors for Portuguese investors. Small and medium enterprises (SMEs), due to their flexible conditions that allow changes in the activity, and the strong trend towards outsourcing, to the detriment of the manufacturing industry, are the primary focus of international investment. This trend, although common to several sectors, has shown greater dynamism in the banking and financial sector.

**Conclusions:** The results suggest market-oriented investments aiming at growth and expansion. The vast Polish market is the one that most attracted Portuguese investors. The hybrid feature of some strategies can align with the cautious attitude towards the investment translated into cooperation agreements with financial institutions for funding, the market learning process, and the training of the personnel. The anticipation of the installation over potential competitors, the experience in production and international markets, the price-quality ratio, the capacity of product adaptation and the design were considered important sources of competitive advantage that motivated the investment. The greatest difficulties during this process were language and the complexity of legislation.

## Introduction

It was not until the 1960s, with the great dynamism of North American Multinational Corporations (MNCs) with diverse competitive strategies, that the role of foreign investment has been highlighted in endogenous growth models, combined with studies on technology diffusion ( Vázquez-Barquero, 2002). The role of Foreign direct investment (FDI) was further highlighted by the performance of some Asian countries in the 1990s as a magic ingredient to economic growth ( Saleem & Shabbir, 2020. Yet, the Asian crisis of 1997/1998 uncovered the weaknesses of the Asian model ( Chung, 2021), leading to a general awareness that the path of sustainable growth involves internalizing the specificities of each economy ( Lin, 2011).

Notwithstanding, currently it is consensual that the preservation of competitiveness involves, in many cases, the international relocation of certain stages of the production process. In a context of open economies, internationalization can become a matter of survival ( Freixanet & Renart, 2020). Yet, large investments in machinery and equipment are not a guarantee of increases in productivity and competitiveness, due to insufficient mastery of organizational aspects ( Shlafman, Bondarenko & Zakcharov, 2020). In addition, inadequacies in terms of strategic reflection and market interpretation can hamper the competitiveness of firms ( Dixit, Singh, Dhir & Dhir, 2021). Also, the sole focus on the production process, rather than considering product design and marketing aspects, can be detrimental to the internationalization process ( De Beule, Van Assche & Nevens, 2022; Sørensen & Ngoc, 2021).

This paper uses interviews and surveys applied to managers of different sectors to carry out 6 case studies to characterize the Portuguese direct investment (PDI) in Poland, Hungary, and the Czech Republic, regarding the determinant factors of attraction, PDI motives, goals, strategies adopted, entry modes, competitive advantages, threats, and challenges.

We assume the entrepreneurs decide to invest in those countries for the business opportunity, being fundamentally attracted by the quality-price ratio of the workforce.

## FDI: Motivations, entry modes and objectives

The competitive advantages of firms may arise from the holding pioneering positions in the implementation of the appropriate strategy and/or motivation to produce and innovate, determined by the nature of their customers and competitors and by the use of resources and productivity ( Ferreira, Coelho & Moutinho, 2020) In this process, the environment is particularly important, as it allows for a rapid accumulation of resources and specialized skills and guarantees access to information on market needs ( Mostafiz, Sambasivan & Goh, 2019) Thus, the analysis of the competitive advantages and corporate strategies to address the internationalization process, should reflect the interaction of six variables: the government, which should act as a catalyst for change, encouraging firms to become more competitive, monitoring compliance quality standards and regulating competition; the firm (structure, strategies and competition) that should stimulate the creation and maintenance of competitive advantages, which may translate into innovation, improved efficiency, cost reduction and improved product quality; the production factors that must be specialized and meet firm’s needs, otherwise strong competitive positions will be created; the internationally related competitive sectors that push for the increase of global competitiveness; and the demand that allows understanding the market’s needs and can induce innovation; and chance (facts beyond firm’s control, such as wars, technological progress, political events, etc.) that may stimulate firms’ competitiveness. Companies that internationalize should develop a strategy to disseminate activities in the value chain, to enhance their sources of competitive advantage ( Chen, 2018; Porter, 1986). In this context, downstream activities will be typically located in the host country, while upstream activities may be in the home country. Hence, downstream activities may create host country specific competitive advantages, such as low costs and product differentiation.

The different ways in which the presence of firms in the foreign market is reflected depend on the type of competitive advantage they hold and are distinguished by the configuration of activities (dispersed/concentrated) and their coordination (weak/strong) ( Feio, 1998; Hernández & Pedersen, 2017).

**Motivations for FDI** can be the search for resources, strategic assets, technology, markets, and diversification. The first aims at exploit natural resources to obtain/secure an uninterrupted supply that allows for cost reduction. The second aim at maximizing the firm’s overall performance. The third is related to access to sophisticated technologies and know-how. In some countries, markets may be saturated and thus firms need to target markets abroad to sell their products. In this process, firms may face trade barriers and chose to invest abroad to overcome such barriers. Finally, particularly large, and relatively competitive firms in international markets, may engage in overseas’ investments aiming at risk diversification.

Among export solutions (agent/exporter, distribution subsidiary) and foreign direct investment (Mergers & Acquisitions [M&A], Greenfield, joint venture), there several entry modes in the foreign markets, such as collaboration with local companies to take advantage of their knowledge of the foreign market (licensing, franchising, technical agreements, management contracts, strategic alliances) which reduce penetration costs, but may lead to imitation/appropriation by local rivals.

The choice between export and FDI will be made considering internal and external aspects of the company. Exports are facilitated by technological developments and international trade regulations that contribute to reductions in transport, telecommunications, and tariffs, and to tax harmonization, and reduce the interest in diversifying production units (FDI). If there are obstacles to exports, companies may use agents or local importers to offer their products (Arm's length). However, there are products and services that, by their nature, are difficult to supply in this way, namely when it is necessary to provide an after-sales service, adaptation of products, or when there is a strong component of brand reputation. Thus, many cases of vertical and horizontal integration resulted from the need to prevent problems arising from uncertainty, or increased costs arising from commercial mechanisms (which may result from geographic or cultural distance, differences in the legal framework or institutional dimension). In short, factors such as tariffs and quotas, discrimination against foreign companies, transport costs, distance, strong competition, high technology, and experience in foreign markets tend to favor foreign direct investment. On the contrary, exports are favored by restrictions on the possession of foreign capital and by political and exchange rate risks.

An underlying issue of the internationalization process is its genesis, since, in some cases, the internationalization process is the result of chance, although typically involves a deliberate strategy. Each internationalization strategy can be described as the association between some important internal advantages of the company (I) and external factors (E), where:

KH - Know-how, knowledge, and accumulated experience.

MF - Available financial means (internal and external).

SM - Access to Markets.

CP - Installed production capacity, infrastructures that enable production.

ME - External environment, context in which the company operates, that is likely to motivate FDI (legislation, Government support and incentives, etc.).

Crossing I and E ( Table 1), the main diagonal does not appear be of much relevance since it corresponds to a reinforcement of the advantages that already exist at the home market. The SM column represents the conquest of more attractive markets.

**Table 1.** Matrix of the determinants of FDI.

E	KH	SM	MF	CP	ME
**I**					
**KH**	KH; KH	KH; SM	KH; MF	KH; CP	KH; ME
**SM**	SM; KH	SM; SM	SM; MF	SM; CP	SM; ME
**MF**	MF; KH	MF; SM	MF; MF	MF; CP	MF; ME
**CP**	CP; KH	CP; SM	CP; MF	CP; CP	CP; ME
**ME**	ME; KH	ME; SM	ME; MF	ME; CP	ME; ME

Adapted from Valadares *et al*. (1995) with the introduction of variable ME. KH - Know-how; MF - financial means; SM - Access to Markets; CP - production capacity; ME- External environment.

The CP column means the use of installed capacity abroad associated with home production capacity, know-how, market, financing, and the national environment. In this case, the investment must target host countries with lower costs or underutilization of existing production capacity, due to industrial conversion processes. The KH column is related to access to foreign know-how to leverage the advantages acquired internally. The MF column is related to easier/cheaper financing abroad and the projection of national competences abroad to reduce financing costs. Finally, the ME column represents situations of granting incentives by foreign authorities, favorable legislation to attract investment in the host countries and/or the presence of other national firms in the host country (suppliers, customers, competitors) with which the company has strong links.

**Foreign investment objectives.** Foreign direct investment may aim at commercial expansion or at rationalizing costs using cheaper production factors. Commercial expansion may become the main objective when the capacity of the domestic market is weak or there are high transport costs, restrictions on foreign trade or restrictions imposed by consumers (nationalism, product image, uncertainty of supply or the need to product adaptation) or when firms need to follow their competitors/customers. Investments aimed at taking advantage of production factors are motivated by difficulties in accessing production factors or differences in their cost in the country of origin, rationalization of production, vertical integration, high installed capacity without corresponding demand in the domestic market, incentives or due to the advanced stage of the products’ life cycle. However, currently, these two objectives tend to be diluted, since some host countries have become important both for cost rationalization and as new markets.

## Methods

### Study design and questionnaire

Quantitative research was composed of a survey applied to a non-probabilistic sample of managers. Inclusion criteria were managers of Portuguese firms investing in at least one of the three countries (Poland, Hungary, or the Czech Republic).

The analysis is based on a survey by Bartels *et al*. (2009). The first author distributed the questionnaires and explained them to the target respondents. The completed questionnaires were gathered just before the interviews. Data from questionnaires were introduced into STATA 17.0 for internal consistency analysis. Variables concerning motivation, determinant factors, and barriers were obtained from the literature on international business and FDI ( Castro, 2000; Leahy & Pavelin, 2003; Kim & Rhee 2009; Bartels *et al*., 2009; Kedia *et al*., 2012) and location and transaction cost decisions ( Dunning, 1998). The variables of the survey questionnaire were structured as Likert scale questions.

The questionnaire has 4 groups. The first concerns the identification of the firm, and the second, third, and fourth groups report motivation (37 items), determinant factors of attraction (16 items), and barriers (17 items) to the investment abroad. Data analysis is descriptive. Since initially, we had 70 variables (items), the recommended minimum number of respondents would be 350 to implement factor analysis (at least 5 times more observations than the number of variables). Therefore, this requirement cannot be satisfied for the analyzed dataset. In addition, it is recommended for factor analysis to use large samples (at least less than greater than 100). Our database could not meet this criterion. The reliability of the questionnaire was obtained through the Cronbach’s alpha calculated using Stata 17.0, to validate the questionnaire. Cronbach's alpha is the average of all variability coefficients that result from the different ways to halve the set of evaluators. From the analysis of variance’s point of view, it can be interpreted as the intraclass correlation coefficient. Thus, the alpha value changes according to the population to which the scale is applied ( Streiner, 2003). In general, the minimum acceptable value for the reliability of a questionnaire is α≥0.70; below which the internal consistency of the scale used is considered low. By contrast, an alpha above 0.90 suggests redundancy or duplication, i.e., several items are measuring the same element of a construct; therefore, redundant items must be eliminated. Thus, ideally, alpha values lie between 0.80 and 0.90 ( Streiner, 2003). The value of alpha depends on the number of items on the scale. As the number of items increases, the variance increases systematically. After running the command alpha, the items for groups 2, 3, and 4 were reduced to 19, 10, and 6 items, respectively. The alpha value is 0.87 which indicates high reliability. The questionnaire can be found as *Extended data* ( Santos, 2022c).

Table 2 shows the dimensions of validity of the survey, assessed with hypotheses, evidence and statistical criteria.

**Table 2.** Dimensions of validity assessed with hypotheses, evidence and statistical criteria.

Validation proposition: Use of the questionnaire as a research measure assessing motivation, determinant factors of attraction and barriers to FDI			
Validity dimension	A priori hypotheses	Sources of evidence	Statistical criteria
Construct validity	Motivation, determinant factors, and barriers have an impact on Portuguese firms’ decisions to invest in Poland, Hungary, and Czech Republic.	Literature review	Not applicable
Internal consistency	Subscales will show good internal consistency after final item selection	Cronbach’s α within subscales	Cronbach’s α ≥ 0.7
Content cohesion			

The second component included exploratory semi-structured interviews conducted with (male) managers at companies’ headquarters. Semi-structured exploratory interviews are the most used interview format for qualitative research. More specifically, the first author carried out interviews structured around an initial open question: why did you invest in Poland and/or Hungary and/or the Czech Republic? The following questions emerged from the dialogue between interviewer and interviewee. Interviews were conducted for each individual, lasting approximately 60 minutes. These interviews aimed at obtaining a more thorough understanding of the motivations and difficulties that constrained the investment in Poland, Hungary, and/or the Czech Republic.

### Sample

We identified 28 Portuguese companies (Population) that invest in at least one of the three countries, of which 15 invest in Poland, 10 in Hungary and 7 in Czech Republic. Some firms invest in more than one country. According to information fron the Portuguese Investment Agency, the sectoral and spatial distribution of the known population of firms is displayed in Table 3.

**Table 3.** Sectoral/spatial distribution of Portuguese outward investment.

Sectors	Poland	Hungary	Czech Republic
Food products (10)	X	X	
Textiles (13)		X	
Wearing apparel (14)	X		
Wood (16)		X	X
Other non-metallic mineral products (23)	X		X
Metal products (25)	X		
Electronics (26)			X
Electrical equipment (27)		X	X
Electricity, gas, steam and air-conditioning (35)	X		
Construction (41)	X	X	X
Retail (47)	X	X	X
Telecommunications (61)	X	X	
Financial services (64)	X	X	
Real estate (68)			X
Cartography (71)			X
**Total sectors**	**9**	**8**	**8**

Notes. The Nomenclature of Economic Activitiesindustries (NACE revision 2) codes between parentheses.

Table 3 shows that the manufacturing industries represent 53% of the population. The number of sectors reflect the great diversity of investment areas. Poland captures more sectors. This fact became evident through the interview to the manager of the construction company (that invests in the three countries). Regarding the attractiveness of the three economies, he has stated: “In objective terms, by descending order, the most developed is the Czech Republic, followed by Hungary and Poland, but the country that most matter for us is Poland, because it is the largest.”

A sample of 15 companies investing in those countries was selected for the case studies. The contacts with the managers of these companies were made by mail, aiming at collecting data via a questionnaire about the reasons, attraction factors and challenges during the investment process. Responses were sought in terms of the importance assigned to each item.

### Interviews

In the interviews, the entrepreneur was allowed to speak freely about the investment process, namely about strategies and future expectations.

Since the sample represents only 25% of the population, it is not representative (in terms of size and diversity of activities) which can compromise the reliability of the conclusions. Thus, we can only expect to provide hints on the outward investment process.

However, the validity of the sample justifies three remarks. First, the lack of a secondary data source, would require a case-by-case compilation, requiring a great deal of time and effort that was not possible; Second, the number of firms in the sample is within the average range of cases in other studies ( Braz, 1999; Valadares *et al*., 1995); Third, although the results are conditioned by the small size of the sample, they nevertheless allow for information that is consistent with other studies (e.g., Benan & Estrin, 2000; Döhrn *,* Milton & Radmacher_Nottelmann, 2001; Resmini, 1999), which justifies their presentation and discussion.

Regarding the interviews, the questions were posed according to the way the interviewee handled the topic, so as not to influence the answers. The interviews also allow to carry out a swot analysis of the recipient countries.

### Data analysis

The interviews were recorded and later transcribed, having been subjected to line-by-line analysis, according to the Grounded Theory, with a view to identifying the main concepts and later structuring the logic underlying the investment decision. In general, the interviews reflect the perception of the external environment by those responsible for the investment. Table 4 provides the sectoral distribution of the sample.

**Table 4.** Sectoral distribution of the sample of firms interviewed.

Firm	Sectors
D	Textiles (13)
C	Electrical equipment (27)
A	Construction (41)
F	Retail (47)
B,E	Financial services (64)

Notes. NACE revision 2 codes between parentheses.

### Ethics and consent

Oral consent was provided, and audio recorded, due to process simplification and because the formality of written information was viewed as inappropriate by the respondents. The ethical approval board from UC approved this study in March 2022. The approval board deemed the oral consent to be adequate for the study.

## Results

### Interview results

**Figure 1.**
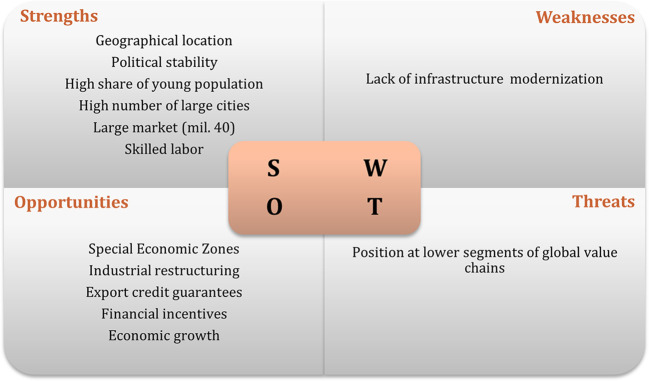
SWOT analysis for Poland.

**Figure 2.**
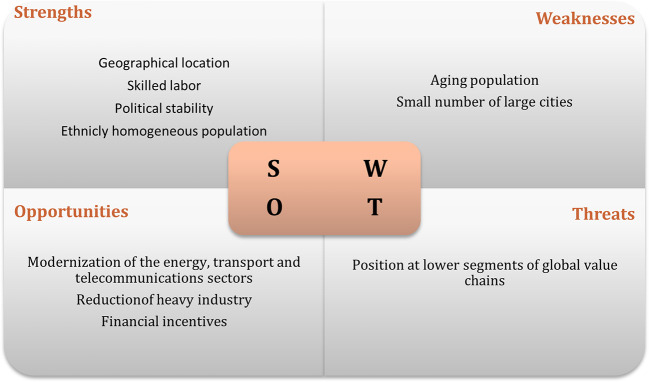
SWOT analysis for Hungary.

**Figure 3.**
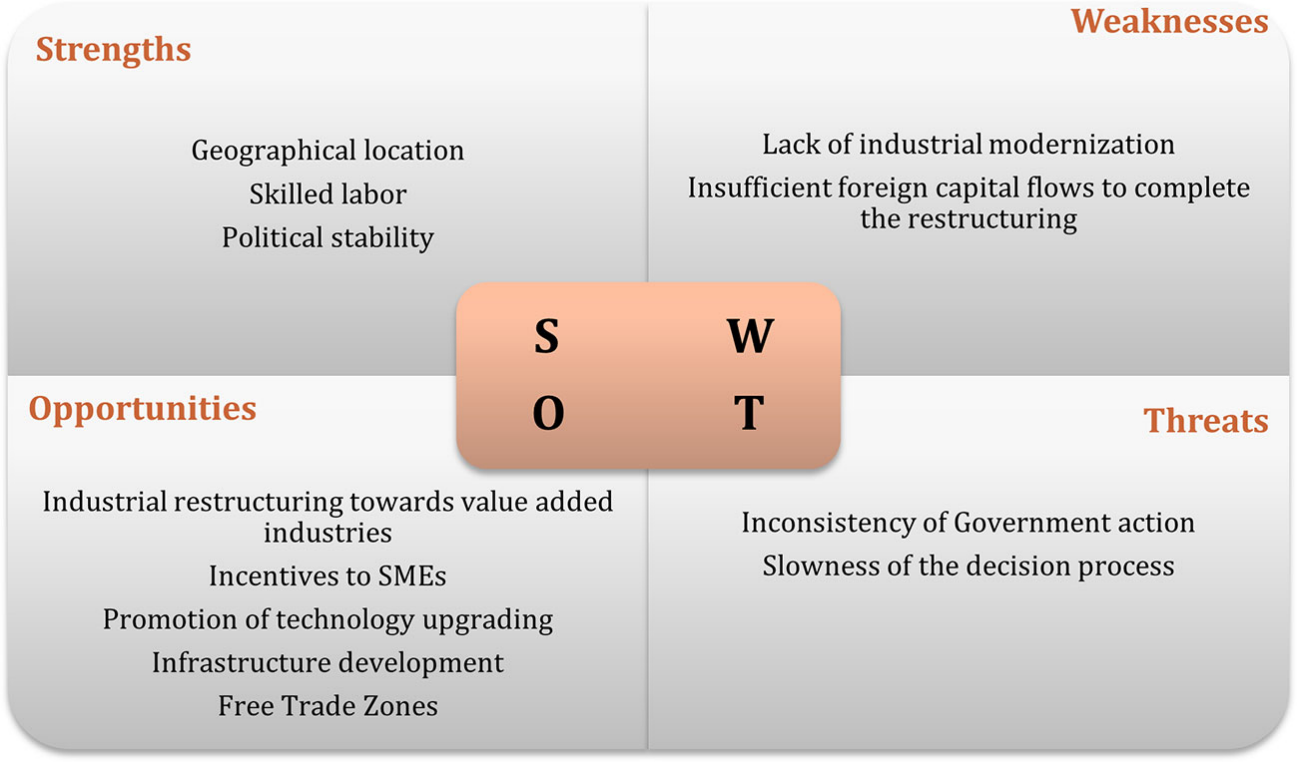
SWOT analysis for Czech Republic.

It was only possible to carry out six interviews, due to the lack of response ( Santos, 2022a). The results of interviews regarding the swot analysis of the recipient countries are displayed in Figures 1 to 3. The figures show that the three economies benefit from common strengths such as geographical location, skilled labor, and political stability.

However, Poland displays six strengths, against five for Hungary and three the Czech Republic. Concerning threats, Poland and Hungary share the same: position and low segments of the global value chain; while the Czech Republic presents as threats, some issues related to the political process.

In relation to weaknesses, the lack of modernization is one of the main and common weakness to Poland and the Czech Republic, while Hungary has an aging population and a small number of cities as main weaknesses. In terms of opportunities, Poland and the Czech Republic have the highest number. Among the most common opportunities are special economic zones, industrial restructuring, financial incentives, and technological upgrading. Thus, the interviews suggest that Poland has the best conditions to attract Portuguese investment, followed by Hungary.

The sample of firms is an example of the ability to project domestic skills on foreign markets. They all have in common the development of active internationalization strategies (with more or less success) aiming various objectives. Table 5 shows the attraction factors, investment motivation, entry mode, objectives and strategy pursued, competitive advantages, threats, and challenges. The most common competitive advantages are installation and anticipation over competitors. Experience, price, and product innovation are listed as not negligible competitive advantages. Companies E and F (financial services and retail) presented the highest number of competitive advantages in relation to the remaining companies in the sample. As for the strategies, the textiles company present a greater variety. The most common strategies for this sample of companies are a cautious attitude towards investment, learning about the market, training the workforce, and investing in the sales team. Firms D and E (textiles and financial services) focused on adapting production to local tastes and introducing new products and concepts that allow customer satisfaction.

**Table 5.** Features of the outward investment of Portugal in the CEECs.

Firms	A	B	C	D	E	F
Competitive Advantages	Installation	Installation Anticipation	Know-how Experience	Good financial situation Experience Product adaptation Design Quality Credibility Price Financial incentives Anticipation	Experience	Anticipation Product differentiation Price-quality ratio Fame and tradition
Strategies	Control Training Market learning Cooperation agreements Cautious attitude	Market studies Training Cautious attitude Price segmentation Commercial Delegations Funding	Transfer of know-how Cautious attitude Local representative Market studies	Large investment Cash and Carry + Show Room Commission agentes Production adaptation Market studies Satisfying different needs Periodic exhibitions	Joint ventures New produtcs and concepts adapted to the local culture	Sales team Expansion
Goals	Expansion	Growth	Profit Survival	Growth	Growth	Growth Cost reduction
Entry modes	M&A	M&A	Joint venture	Greenfield	Joint venture	Greenfield
Motives	Domestic market saturation Overtake competition Risk dispersion Business opportunity	Business opportunity	Domestic market saturation Survival Reduce costs Business opportunity	Commercial network domain Distribution Vertical integration Business opportunity	Globalization Business opportunity Risk diversification Profit Follow customer Coverage of local needs Partenership	Domestic market saturation
Threats	Competition	Credit risk	Little experience and suspicious mindset of partners Appropriation of knowledge	Competition	Competition	Competition
Challenges		Lack of labor Funding	Bureaucracy HRM Language	Economic adaptation phase Charges Funding	Fragmented market Culture	Rent costs Lack of motivation os sales team Transport

Regarding the objectives, Firms C and F (electrical equipment and retail) have a greater number of objectives, which are to regain their presence in the domestic market, profit, survival, cost reduction, as well as sales and growth. The objective of growth is common to all companies. Entry modes are equally represented by M&As in construction and financial services (A, B), joint ventures in electrical equipment and financial services (C, E) and Greenfield projects in textiles and retail (D, F).

As for the motivation for investment, Firm E (financial services) has more motivations such as globalization, business opportunity, risk diversification, profit, covering local needs, following the customer, and establishing partnerships. The most frequent reason is the business opportunity, risk diversification and stagnation of the domestic market.

Regarding the attraction factors, companies A and E (construction and financial services) present the highest number, such as market size, political stability, geographical situation, skilled labor, good professional ethics, high profit margins, economic stability and openness of society and the government. The most common factors are the training of the workforce, the size and growth potential of the market and stability (economic, political, and social). In relation to threats, company C (electrical equipment) presented a greater number, such as the lack of experience and suspicious mentality of the partners and the risk of appropriation of knowledge. The most common threat is competition. In terms of challenges or difficulties, companies C, D and F (electrical equipment, textiles and retail) present a greater number of difficulties such as bureaucracy, human relations, language, low motivation of the selling team, transport/accessibility and the cost of rents. The most common difficulties are related to labor and financing.

Applying the information from the interviews to the Matrix of the determinants of FDI ( Table 1), the results are shown in Tables 6 to 11 and summed-up in Table 12.

**Table 6.** Determinants of FDI for firm A.

E	SM	CP	MF	KH	ME
**I**					
**SM**	A	A			A
**CP**	A	A			A
**MF**	A	A			A
**KH**	A	A			A
**ME**	A	A			A

Notes. KH - Know-how; MF - financial means; SM - Access to Markets; CP - production capacity; ME - External environment.

**Table 7.** Determinants of FDI for firm B.

E	SM	CP	MF	KH	ME
**I**					
**SM**	B	B			B
**CP**	B	B			B
**MF**	B	B			B
**KH**	B	B			B
**ME**					

Notes. KH - Know-how; MF - financial means; SM - Access to Markets; CP - production capacity; ME - External environment.

**Table 8.** Determinants of FDI for firm C.

E	SM	CP	MF	KH	ME
**I**					
**SM**					
**CP**	C	C			C
**MF**	C	C			C
**KH**	C	C			C
**ME**					

Notes. KH - Know-how; MF - financial means; SM - Access to Markets; CP - production capacity; ME - External environment.

**Table 9.** Determinants of FDI for firm D.

E	SM	CP	MF	KH	ME
**I**					
**SM**	D				D
**CP**	D				D
**MF**	D				D
**KH**	D				D
**ME**	D				D

Notes. KH - Know-how; MF - financial means; SM - Access to Markets; CP - production capacity; ME - External environment.

**Table 10.** Determinants of FDI for firm E.

E	SM	CP	MF	KH	ME
**I**					
**SM**	E	E			E
**CP**	E	E			E
**MF**	E	E			E
**KH**	E	E			E
**ME**					

Notes. KH - Know-how; MF - financial means; SM - Access to Markets; CP - production capacity; ME - External environment.

**Table 11.** Determinants of FDI for firm F.

E	SM	CP	MF	KH	ME
**I**					
**SM**	F				F
**CP**	F				F
**MF**	F				F
**KH**	F				F
**ME**					

Notes. KH - Know-how; MF - financial means; SM - Access to Markets; CP - production capacity; ME - External environment.

**Table 12.** Determinants of FDI for the sample of firms.

E	SM	CP	MF	KH	ME
**I**					
**SM**	A+B+D+E+F	A+B+E			A+B+D+E+F
**CP**	A+B+C+D+E +F	A+B+C+ E			A+B+C+D+E+F
**MF**	A+B+C+D+E +F	A+B+C+ E			A+B+C+D+E+F
**KH**	A+B+C+D+E +F	A+B+C+ E			A+B+C+D+E+F
**ME**	A+D				A+D

Notes. KH - Know-how; MF - financial means; SM - Access to Markets; CP - production capacity; ME - External environment.

Firm A (Construction) benefited of its internal advantages (market position, production capacity, financial means, know-how and incentives) to access markets in Poland, Hungary, and the Czech Republic. It also took advantage of external production capacity and the stability and economic development of the foreign markets.

Firm B (Financial services) took advantage of the installed production capacity in Hungary and the legal facilities granted there to access the market. To this end, the firm relied on the good position in the Portuguese market, production capacity, internal financial resources, and know-how.

In the case of firm C (electrical equipment), it was a matter of taking advantage of the production capacity, financial means and know-how acquired internally, to take advantage of the Czech market, as well as the production capacity installed in the host country and its favorable legislation.

For firm D (Textiles) located in Poland, the aim was to access the market to control the distribution channels, taking advantage of the country's environment (mentality, legislation, etc.). As it distributes a product that corresponds to a sector of national production specialization, it was able to gather all the expected internal values (production capacity, prominent position in the national market, financial means, know-how and incentives to internationalization.

Firm E (financial services) has a prominent position in the national market, production capacity, financial means, and know-how. These competitive advantages allowed it to acquire a financial participation on one of biggest polish banks to access the vast market.

Firm F operates in retail in Polish and Hungarian markets, has a stable position at the national market, production capacity, financing and know-how that allowed it to take advantage of external markets and their favorable investment environment.

We can now to integrate the analysis of the six companies in a summary table ( Table 12).

Market-oriented strategy, taking advantage of the environment, is common to all firms. As for internal competencies, all firms possess financial means, know-how, and production capacity, which they designed abroad. Five of them (except for the firm operating in electrical equipment which lost its prominent position in the national market, due to the specific sectoral conditions) consider that the investment had nothing to do with the difficulties of the domestic market.

Firms A and D received incentives to invest abroad, thus we marked the ME item (environment). The two companies operating in the financial sector have entered the foreign market via M&As and joint ventures, thus we marked production capacity (CP) abroad. Also, the construction company acquired a local company, and the electrical equipment company formed a joint venture, with the future ambition of building a factory in the Czech Republic, hence we marked production capacity (CP) abroad.

There are generally five motivations for firms to invest abroad: to gain resources, strategic assets, technology, markets, and diversification. The division is not always straightforward; small and medium enterprises (SMEs) can pursuit multiple objectives by investing abroad. Moreover, the motivations for FDI might change over time, as firms gain experience in the foreign markets. The interviews revealed that Half of the analyzed sectors followed the single internationalization strategy of market seeking investments (construction and retail); whilst firms operating in financial services and electrical equipment adopted a mix of strategies.

### Surveys’ results

It was only possible to carry out seven surveys, due to the lack of response ( Santos, 2022b). We calculated the medians of the Likert scale for each item/group. Starting with Motivation (group 2), among the main reasons for investing in those countries are marketing advantages, the importance of the foreign market in terms of turnover and profit. These findings are related to the nature of the sample of sectors and because most companies invest in Poland, the larger market of all three. Indeed, investments directed to Poland are mostly market seeking, with a view to supply the neighboring countries.

Motivations such as the need to reduce the costs of supplying foreign markets, adapting products, counterattacking foreign rivals, reducing information costs, mitigating the uncertainty of the supply, eliminate transport costs, improve product quality, overcome non-tariff barriers or nationalism were not considered relevant by the respondents. This suggest that the investment was not motivated by difficulties in the market of origin or arising from exports, rather fit into a logic of supplying local markets.

**Figure 4.**
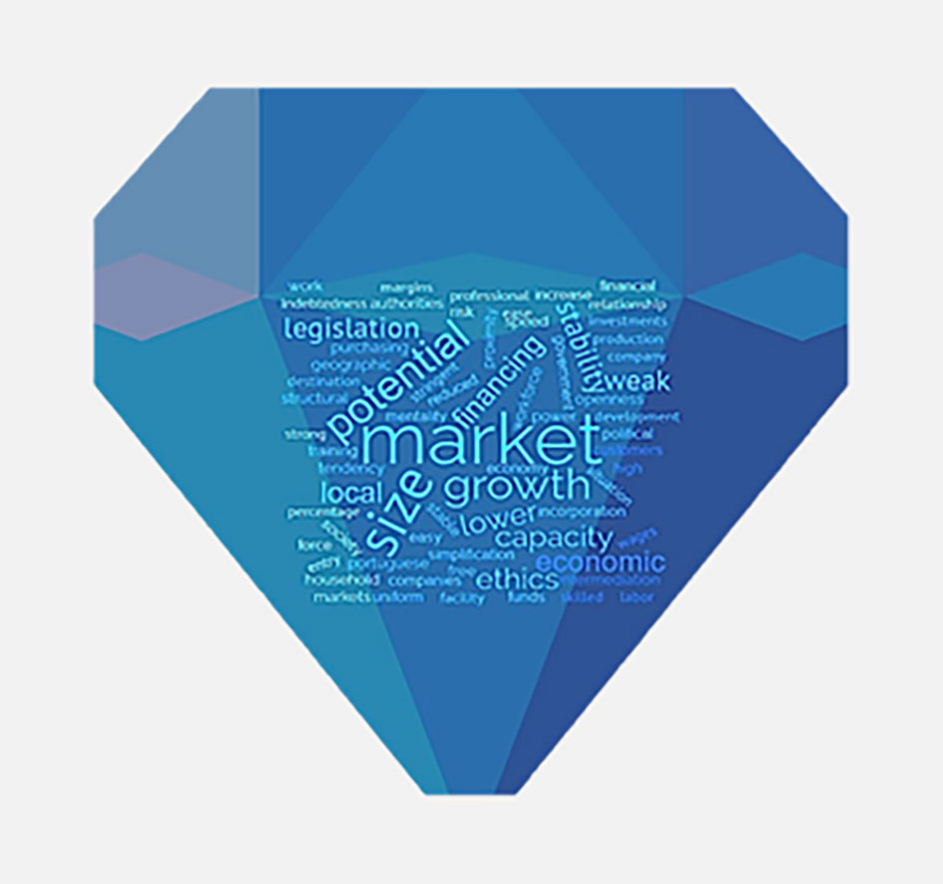
Factors of attraction of Portuguese direct investment in the Central and Eastern European Countries.

The main attraction factors are the distribution channels, the size and the growth potential of host countries’ markets, the institutional framework, and the reduced political risk ( Figure 4).

The wages in host countries were not considered relevant. Resmini (1999) comes to the same conclusion when stating that the low cost of labor is not an important objective for the FDI, given that it is oriented towards the long run when wage differentials tend to disappear.

Other authors highlighted the fact that, once the location decision has been made, in a country with low labor costs, the search for labor at the lowest possible cost may or may not be important. Natural resources are considered non-relevant for the investment, since none of the companies adopted a resource seeking strategy.

The survey’s results indicate that the role of government regarding financial incentives has been redundant for the investment decision. This is in line with the idea that investments must be profitable per se and not rely on state aid. In addition, the changing nature of incentives over time may introduce an element of mistrust among investors. Language and the existence of complex legislation were mentioned as the main challenges/difficulties that may occur in the investment process. However, culture is not considered a major challenge, which can be explained, in part, by openness of society in those countries. Real estate and financing were not considered difficulties since the main forms of market penetration were M&As and joint ventures, and Portuguese banks appear to be following their clients to these countries.

### Results by sector

*Retail (textiles and footwear)*

Given the advanced stage of the product cycle, investment was aimed at commercial expansion, considering the need to exceed limits on quantities to be sold and to diversify activities. In this process, finding niche markets was considered of secondary importance. Thus, the market and distribution channels were important for entrepreneurs. The investment was not motivated by the need to follow competitors or to counterattack them in their own country. Moreover, the investment was not aimed at taking advantage of production factors (resources, labor) and was not determined by the need to follow customers or by restrictions imposed by consumers (nationalism, product image, uncertainty of supply or the need to adapt products) or to overcome exports’ issues (e.g., non-tariff barriers).

*Financial sector (banking and credit institutions)*

The reasons for the investment were mostly related to the business opportunity, due to the advanced stage of the product cycle. The need to increase competitiveness through economies of scale and service efficiency were considered secondary. Competition and resources played a small role in investment’s decisions. Managers considered that there was no need to improve product quality. The reduced political risk and the institutional framework were the main factors of attraction, while the quality of labor and good infrastructures were considered with medium importance.

## Discussion

Our results corroborate the findings of several authors. First, Market size is an important attraction factor for FDI ( Azam & Lukman 2010; Curran *et al*., 2017). Second, there is no evidence that FDI was induced by tariffs in the host countries ( Agodo, 1978; Moore, 1993). Third, human capital is less important than the market for investment decisions in developing countries ( Deyo, 1989). Fourth, political events (e.g., nationalization of foreign-owned assets) can disrupt the economies by jeopardizing past investments ( Castro, 2000). Bartels *et al*. (2009) showed that FDI in ten Sub-Saharan African countries was mostly motivated by political economy considerations, rather than by labor and production input variables. Also, Institutional factors (good government governance, economic freedom, public efficiency) play an important role in FDI decisions ( Dixit, 2011). Tomelin *et al*. (2018) and Minh (2019) conclude that institutional quality (legal structure, strong property rights, freedom to trade, and civil liberties) is important for FDI attraction. Gubik *et al*. (2020) highlighted tax optimization, geographical distance, and global production chain as major motivations for FDI in the Visegrad countries (Czech Republic, Hungary, Poland, and Slovakia). Sixth, incentives were not a relevant determinant of FDI ( UNCTAD, 1998).

By contrast, we could not find evidence to support the findings of several authors. First, contrary to Castro (2000), the incentives to foreign investors were not important instrument of investment policies. Second, we could not support the idea that the availability of raw materials and the cost and labor supply have a major impact on FDI decisions as highlighted by Dunning (1988). The same happens for the transport costs pointed out by Dunning (2015). Also, Tsai (1994) found that lower labor costs are important to attract FDI to economies. Third, we could not support the argument that culture is the core motivations for FDI, unlike Saleh *et al*. (2017). Fourth, Hirsch, *et al*. (2020) found that water resources and land abundance are major determinants of FDI contrary to our results.

Unlike Kedia *et al*. (2012) we could not support the point of view that many firms start as niche providers for their rivals that are already established in the market, and as their role becomes more important in these firms’ value chain, they may aim at acquiring the ability to compete in product development abroad.

Also, unlike Leahy & Pavelin (2003) we did not find that domestic competitors have followed the leader abroad to facilitate collusive behavior in the markets in which they compete. In manufacturing and service industries, firms may follow their customers abroad to keep them ( Kim & Rhee, 2009). However, that was not the case of managers that were interviewed. Finally, the quality of the workforce is pointed as another factor of attraction of FDI ( Chansomphou & Ichihashi, 2011). Chakraborty *et al*. (2018) states that labour efficiency is more important to attracting foreign firms than Infrastructure, in India. We could not corroborate these findings.

The viability of the internationalization model requires the analysis of the main agents that compete for its implementation: consumers, competitors, foreign capital, and national and foreign authorities. Although each agent can intervene differently, depending on their objectives and constraints, some dominant behaviors can be typified: consumers and national authorities are receptive to FDI; competitors consider it a threat; and foreign authorities are faced with a dilemma of defending domestic firms/attracting FDI. In this framework, industrial policy plays an important role by contributing to the achievement of higher levels of competitiveness through the increase of manufacturing productivity ( Santos & Khan, 2019). Results suggest that investments were market-oriented aiming at expansion and profit. This requires firms’ competitiveness, which involves preserving the economic sustainability of firms ( Santos & Moreira, 2022). In this context, the vast Polish market was the one that most attracted Portuguese investors. The lower GDP per capita in Poland compared to the other two economies, suggests that the size of the potential market overcame consumer purchasing power considerations in the investment decisions. State aid, in turn, only played a supporting role in the investment, not constituting, in any case, the engine of that process. As the manager of company D said: “… no investments were made because of incentives. Many people invest because they will have support. This does not work. Investments must make sense per se. Investments should be viable or else, one can have all support in the world, and it still do not work. Off course, the support is very welcome, and sometimes can leverage these projects to succeed and consolidate faster.”

The hybrid feature of some strategies can align with the cautious attitude towards the investment (risk aversion), translated into cooperation agreements with financial institutions for funding, the market learning process, and the training of the personnel.

The anticipation of the installation over potential competitors, the experience in production and international markets, the price-quality ratio, the capacity of product adaptation and the design were considered important sources of competitive advantage that motivated the investment. The greatest difficulties during this process were language and the complexity of legislation.

*Limitations.* As mentioned above, the small number of respondents makes it difficult to use modern statistical methods of analysis such as factorial analysis. We carried out a summary of the content of interviews without using qualitative software (e.g., Maxqda, NVivo, etc.). Since the sample represents only 25% of the population, it is not representative (in terms of size and diversity of activities) which can compromise the reliability of the conclusions. Thus, we can only expect to provide hints on the outward investment process. Also, the analysis of the matrix of determinants of FDI is characterized by an excessive simplification that can be overcome through a multiple analysis, combining, in each column or row, several internal advantages/external attraction factors. Due to the importance given to the investment process in unknown foreign environments, it seems more realistic to admit that firms only decide to invest abroad when there are strong competitive advantages to offset the disadvantages or when there is more than one internal advantage and/or external attraction factor.

## Conclusion

Despite the business opportunities, Portuguese investment directed towards Poland, Hungary, and the Czech Republic is negligible, due, in part, to the geographic and cultural distance. However, the economic and political stability, combined with market size and growth potential are undeniable attraction factors for Portuguese investors. SMEs, due to their flexible conditions that allow changes in the activity, and the strong trend towards outsourcing, to the detriment of the manufacturing industry, are the primary focus of international investment. This trend, although common to several sectors, has shown greater dynamism in the banking and financial sector.

On the other hand, the decline of large heavy industries, with strong investments in physical capital, led to the adoption of diversification strategies, with a focus on new products and technologies, namely related to information processing, telecommunications, and robotics.

This reformulation of the organization and business strategies, with a view to strengthening the competitive position, may involve the establishment of networks by Portuguese companies, with a view to exploiting the different production conditions in the CEECs.

The accession to the European Union (EU) has facilitated the capacity of these countries to attract FDI. However, the small size of domestic markets, in the case of Hungary and the Czech Republic may reduce their attractiveness for market-seeking investments in case the supply is more profitable via imports. This would lead to divestments resulting from internal productive reorganization.

The qualitative and quantitative evolution of outward Investment from Portugal will depend on investors' motivations and the conditions offered to them. These motivations are influenced by several factors, namely, the evolution of competition on an international scale, the growing competition between countries in attracting investments and the qualitative level of infrastructure to support economic activity in host countries.

The potential positive effects of FDI on host countries, namely on employment, resource transfer and on the external accounts could launch these economies on a growth path. From the home country perspective, activities abroad, in addition to providing obvious benefits at the microeconomic level, may generate direct gains in terms of the balance of payments, and indirect gains, in terms of growth and development. Thus, Portuguese investment in the CEECs can be a win-win game.

## Data availability

### Underlying data

Figshare: Interviews. Challenges https://doi.org/10.6084/m9.figshare.20057027.v1 ( Santos, 2022a).

This project contains the following underlying data:

- Interviews.pdf (interview transcripts)

Figshare. Dataset. https://doi.org/10.6084/m9.figshare.20433141 ( Santos, 2022b).

- Raw responses.csv (responses to survey)

### Extended data

Figshare: Questionnaire. https://doi.org/10.6084/m9.figshare.20358987.v1 ( Santos, 2022c).

- Questionnaire.pdf

Data are available under the terms of the Creative Commons Attribution 4.0 International license (CC-BY 4.0).
