# Outward investment of Portuguese small and medium enterprises in the Central and Eastern European countries: motivations and challenges

**DOI:** 10.12688/f1000research.122697.3

**Published:** 2024-05-31

**Authors:** Eleonora Santos, Jacinta Moreira

**Affiliations:** 1 Centre of Applied Research in Management and Economics, Polytechnic Institute of Leiria, Leiria, 2411-901, Portugal

**Keywords:** Outward foreign direct investment, CEECs, Sttategies, Internationalization

## Abstract

**Background:**

This paper identifies the determinant factors of Portuguese investment in Poland, Hungary, and the Czech Republic. We assume that investment abroad is motivated by business opportunities, and the quality-price ratio of the workforce.

**Methods:**

To this end, we used a qualitative methodology composed of 6 case studies, based on interviews and surveys with the managers of the Portuguese firms investing in those three economies.

**Results:**

Despite the business opportunities, Portuguese investment directed towards these economies is negligible, due, in part, to the geographic and cultural distance. However, the economic and political stability, combined with market size and growth potential are undeniable attraction factors for Portuguese investors. Small and medium enterprises (SMEs), due to their flexible conditions that allow changes in the activity, and the strong trend towards outsourcing, to the detriment of the manufacturing industry, are the primary focus of international investment. This trend, although common to several sectors, has shown greater dynamism in the banking and financial sector.

**Conclusions:**

The results suggest market-oriented investments aiming at growth and expansion. The vast Polish market is the one that most attracted Portuguese investors. The hybrid feature of some strategies can align with the cautious attitude towards the investment translated into cooperation agreements with financial institutions for funding, the market learning process, and the training of the personnel. The anticipation of the installation over potential competitors, the experience in production and international markets, the price-quality ratio, the capacity of product adaptation and the design were considered important sources of competitive advantage that motivated the investment. The greatest difficulties during this process were language and the complexity of legislation.

## Introduction

The exploration of foreign investment gained prominence in the 1960s, propelled by the dynamism of North American Multinational Corporations (MNCs) and their varied competitive strategies, as highlighted by Vázquez-Barquero (2002). Further emphasizing the significance of Foreign Direct Investment (FDI), the 1990s witnessed the performance of certain Asian countries, considering it a catalyst for economic growth, as discussed by Saleem and Shabbir (2020). However, the Asian crisis of 1997/1998 revealed vulnerabilities in the Asian model ( Chung, 2021), prompting a realization that sustainable growth entails internalizing the specificities of each economy ( Lin, 2011).

Acknowledging the consensus that preserving competitiveness often involves the international relocation of production stages, this study recognizes the critical role of internationalization in the survival of open economies ( Freixanet & Renart, 2020). However, it cautions against assuming that substantial investments in machinery and equipment guarantee enhanced productivity and competitiveness, stressing the need for a comprehensive understanding of organizational aspects ( Shlafman, Bondarenko & Zakcharov, 2020). Moreover, strategic reflection and market interpretation are vital for a holistic approach to competitiveness, emphasizing that a singular focus on the production process may be detrimental to internationalization ( De Beule, Van Assche & Nevens, 2022; Sørensen & Ngoc, 2021).

In this study, we adopt a comprehensive theoretical framework that encompasses various perspectives from international business and investment theories. The Resource-Based View (RBV), rooted in strategic management, plays a central role in understanding the internationalization processes of firms in Portugal. RBV emphasizes the distinct set of resources and capabilities that drive competitive advantage and performance. We explore firm-specific resources, including technological capabilities, skilled human capital, networks, and proprietary knowledge, to discern the factors that differentiate Portuguese firms in the global market. Transaction Cost Economics (TCE), proposed by Williamson, sheds light on challenges in international business, specifically addressing language barriers and legislative complexity. TCE provides insights into how these challenges influence the mode of entry and the decision to internalize operations. In the context of Portuguese companies investing in Central European countries, TCE helps us understand the impact of language barriers and complex legislation, offering practical implications for mitigating transaction costs associated with unfamiliar legal frameworks and languages. The Eclectic Paradigm (OLI Framework), introduced by Dunning, serves as another foundational theory for understanding foreign direct investment (FDI). It emphasizes ownership advantages, favorable location, and internalization of operations. For Portuguese SMEs, the OLI Framework provides insights into why firms choose FDI over alternative entry modes. Economic and political stability, market size, and growth potential in Poland, Hungary, and the Czech Republic act as critical location factors influencing Portuguese SMEs’ decisions to internalize operations.

These theoretical foundations contribute to our exploration of Portuguese Direct Investment (PDI) in Central European countries. The study aims to elucidate key aspects such as determinant factors of attraction, PDI motives, goals, strategies, entry modes, competitive advantages, threats, and challenges. In the context of SMEs, the research aligns with the unique needs and concerns of Portuguese businesses seeking international expansion. By addressing motivations, entry modes, and competitive dynamics, the study provides valuable insights for SMEs in Portugal considering or engaged in internationalization, particularly in Central European markets. It offers practical implications for optimizing operations, understanding competitive advantages, and navigating challenges in foreign markets, ultimately aiding SMEs’ preparedness for successful international ventures.

Small and Medium-sized Enterprises (SMEs) play a vital role in shaping Portugal’s business landscape. They constitute the majority of businesses, forming the backbone of the nation’s economic structure. SMEs significantly contribute to the Gross Value Added (GVA) of the country, reflecting their substantial role in creating economic value through the production of goods and services. These enterprises are essential for Portugal’s economic development, impacting key areas such as employment, innovation, and overall economic growth. However, SMEs encounter distinct challenges, particularly when expanding into international markets, where limited resources expose them to potential threats from multinational corporations and larger competitors. Recognizing their significance, the Portuguese government has launched initiatives, exemplified by Portugal 2020, aimed at supporting SMEs in their internationalization endeavors. These programs include financial incentives, information sharing, and comprehensive assistance to enhance the global competitiveness of SMEs. In essence, the success of SMEs is pivotal for Portugal’s economic advancement and long-term sustainability, and government initiatives strive to fortify their position in the global business arena.

This paper aims to enhance the understanding of Portuguese Direct Investment (PDI) in Poland, Hungary, and the Czech Republic by conducting interviews, surveys, and six case studies across diverse sectors. The research focuses on elucidating key aspects such as determinant factors of attraction, PDI motives, goals, strategies, entry modes, competitive advantages, threats, and challenges. The underlying assumption is that entrepreneurs invest in these countries due to business opportunities, particularly driven by the quality-price ratio of the workforce. The study’s relevance for SMEs in Portugal is evident as it provides valuable insights for those considering or engaged in internationalization, specifically in Central European markets. By addressing motivations, entry modes, and competitive dynamics, the research aligns with the unique needs and concerns of Portuguese SMEs. It offers practical implications for SMEs seeking international expansion in Central Europe, helping them understand the factors influencing successful direct investments. The examination of competitive advantages and potential threats contributes to SMEs’ effective navigation of challenges, ultimately aiding their preparedness for international ventures. Moreover, the research contributes to the broader discourse on SME internationalization, offering insights that can be translated into actionable strategies for optimizing operations and competitiveness in foreign markets, providing practical implications for Portuguese SMEs in the investigated regions.

## Theoretical background

This study draws upon theories within international business and investment, providing a comprehensive framework to understand the determinants and motivations behind Portuguese investments in Poland, Hungary, and the Czech Republic. Each theory contributes a unique perspective, shedding light on different facets of the investment decision-making process.

***Eclectic Paradigm (OLI Framework):*** The Eclectic Paradigm ( Dunning, 1980, 2000), also known as the Ownership, Location, and Internalization (OLI) Framework, serves as a foundational theory for understanding foreign direct investment (FDI). Proposed by John Dunning, this framework suggests that firms engage in FDI when they possess specific ownership advantages (O), find a favorable location (L), and decide to internalize operations rather than relying on external markets. Ownership advantages may include unique capabilities, technologies, or brand reputation. Location factors encompass the economic and political stability of the host country, market size, and growth potential. Internalization occurs when firms choose to control foreign operations to safeguard their advantages. In the context of Portuguese investments in CEE countries, the Eclectic Paradigm helps elucidate why firms opt for FDI rather than alternative modes of entry ( Dunning, 1980). Economic and political stability, market size, and growth potential in Poland, Hungary, and the Czech Republic act as critical location factors, influencing Portuguese firms’ decisions to internalize operations. These factors are pivotal in gaining insights into the motivations and strategies driving Portuguese investments in the region.

***Uppsala Model:*** The Market Attractiveness Theory ( Johanson *et al.*, 1975; Johanson & Vahlne, 1977), closely associated with the Uppsala Model, posits that firms consider the economic and political stability of foreign markets when making investment decisions. The Uppsala Model, developed by Johanson and Vahlne, emphasizes the incremental and experiential nature of internationalization. According to this model, firms gradually increase their commitment to foreign markets as they accumulate knowledge and experience. For Portuguese investments in CEE countries, the Market Attractiveness Theory and the Uppsala Model offer insights into the gradual expansion of firms into these markets ( Johanson & Vahlne, 1977). Economic and political stability, as well as the size and growth potential of the CEE economies, act as crucial factors influencing the attractiveness of these markets. Portuguese firms are likely to follow a step-by-step internationalization process, starting with markets perceived as less risky and gradually expanding to more complex environments. Thus, we suggest that:

**H1 -** Portuguese investments in Poland, Hungary, and the Czech Republic are influenced by the economic and political stability, as well as the size and growth potential of the markets in these countries.

**Motivation for FDI Theory:** Motivation for FDI theory ( Rugman& Verbeke, 1998; Caves, 1996) posits that firms invest abroad to capitalize on identified business opportunities and leverage the quality-price ratio of the workforce. Internalization theory complements this perspective by suggesting that firms opt for foreign investments when the advantages of internalizing operations outweigh the reliance on external markets. In the case of Portuguese investments in CEE countries, firms are motivated by specific business opportunities present in these markets. These opportunities may include access to a larger consumer base, favorable regulatory environments, or gaps in the market that Portuguese firms can exploit. Additionally, the quality-price ratio of the workforce in CEE countries may serve as a motivating factor, especially if firms can achieve cost efficiencies without compromising on quality. Thus, we posit that:

**H2 -** Portuguese investment in these economies is primarily motivated by identified business opportunities and the quality-price ratio of the workforce.

Internalization theory complements this perspective by emphasizing that firms opt for foreign investments when internalizing operations provides advantages over relying solely on external markets.

**Resource-Based View (RBV):** The Market-Oriented Investment Strategy aligns with the RBV ( Wernerfelt, 1984; Barney, 1991), suggesting that firms deploy their unique resources and capabilities in international markets to achieve a competitive advantage. In the context of Portuguese investments in CEE countries, this theory implies that firms leverage their internal strengths to succeed in foreign markets. Portuguese firms adopting a market-oriented strategy in CEE countries focus on growth and expansion. They align their resources, such as technological capabilities, brand reputation, and market knowledge, with the demands and opportunities present in the CEE markets. The RBV offers a lens to understand how Portuguese firms strategically position themselves to gain a competitive edge in the region. Therefore, we hypothesize that:

**H3 -** Portuguese firms adopt market-oriented strategies, directing their investments towards growth and expansion in the markets of Poland, Hungary, and the Czech Republic.

**Competitive Advantage Theory (Porter’s Diamond Model):** Competitive Advantage Theory ( Porter, 1985, 1990), rooted in Porter’s Diamond Model, emphasizes the role of firm-specific advantages in enhancing international competitiveness. The Diamond Model identifies factors such as factor conditions, demand conditions, related and supporting industries, and firm strategy, structure, and rivalry as determinants of competitive advantage. For Portuguese investments in CEE countries, the Competitive Advantage Theory posits that the anticipation of installation, experience, price-quality ratio, and product adaptation are crucial sources of competitive advantage. Portuguese firms leverage these factors to position themselves favorably in the CEE markets, enhancing their competitiveness in the region. Hence, we suggest that:

**H4 -** Anticipation of installation, experience in production and international markets, price-quality ratio, and product adaptation are considered crucial sources of competitive advantage driving Portuguese investments in these economies.

**Transaction Cost Economics (TCE):** Challenges in International Business, informed by TCE ( Williamson, 1979; Teece, 1986), acknowledges that language and legislative complexity pose significant hurdles for firms venturing into foreign markets. TCE provides insights into how challenges related to information asymmetry and contracting influence the mode of entry and the decision to internalize operations. In the case of Portuguese investments in CEE countries, language barriers and complex legislation emerge as challenges. These challenges may affect the choice of entry mode, with firms opting for internalization to mitigate transaction costs associated with navigating unfamiliar legal frameworks and languages. Thus, our last hypothesis is:

**H5 -** Language and the complexity of legislation pose significant challenges during the investment process for Portuguese firms in Poland, Hungary, and the Czech Republic.

This theoretical framework integrates perspectives from the Eclectic Paradigm, Market Attractiveness Theory (Uppsala Model), Motivation for FDI Theory, Market-Oriented Investment Strategy (RBV), Competitive Advantage Theory (Porter’s Diamond Model), and Challenges in International Business (TCE). By synthesizing these theories, the study aims to provide a robust foundation for hypothesis development and empirical investigation into the intricate dynamics of Portuguese investments in CEE countries.

## FDI: Motivations, entry modes and objectives

Firm competitiveness is shaped by pioneering positions in strategy implementation and the motivation to innovate, guided by customer and competitor dynamics, resource utilization, and productivity ( Ferreira, Coelho & Moutinho, 2020). In this context, the environmental impact, rapid resource accumulation, and skill development are acknowledged, incorporating insights from Mostafiz, Sambasivan & Goh (2019). This study adopts a framework incorporating government catalyst roles, firm strategies, production factors, competitive sectors, market demand, and external factors beyond the firm’s control ( Chen, 2018; Porter, 1986). Emphasizing the dissemination of activities in the value chain, downstream activities are situated in the host country, creating specific competitive advantages like low costs and product differentiation.

The presence of firms in foreign markets reflects diverse competitive advantages, distinguished by the configuration and coordination of activities ( Feio, 1998; Hernández & Pedersen, 2017). Motivations for FDI encompass resource exploitation, maximizing overall performance, and accessing sophisticated technologies and know-how ( Nguyen *et al.*, 2023). Market saturation in certain countries may drive firms to target overseas markets, necessitating FDI to overcome trade barriers. Large, competitive firms may engage in overseas investments for risk diversification ( Sarker & Serieux, 2023).

Various entry modes, including licensing, franchising, technical agreements, management contracts, and strategic alliances, are explored for their role in reducing penetration costs, although they may expose firms to imitation by local rivals ( Nguyen *et al.*, 2023). The choice between export and FDI considers internal and external factors. Technological developments and international trade regulations favor exports ( Eicher, Helfman & Lenkoski, 2012), while factors such as tariffs, discrimination, transport costs, and strong competition tend to favor foreign direct investment. This dynamic is further shaped by restrictions on foreign capital possession and political and exchange rate risks.

Motivations for FDI can be the search for resources, strategic assets, technology, markets, and diversification. The first aims at exploit natural resources to obtain/secure an uninterrupted supply that allows for cost reduction. The second aim at maximizing the firm’s overall performance. The third is related to access to sophisticated technologies and know-how. In some countries, markets may be saturated and thus firms need to target markets abroad to sell their products. In this process, firms may face trade barriers and chose to invest abroad to overcome such barriers. Finally, particularly large, and relatively competitive firms in international markets, may engage in overseas’ investments aiming at risk diversification.

Among export solutions (agent/exporter, distribution subsidiary) and foreign direct investment (Mergers & Acquisitions [M&A], Greenfield, joint venture), there several entry modes in the foreign markets, such as collaboration with local companies to take advantage of their knowledge of the foreign market (licensing, franchising, technical agreements, management contracts, strategic alliances) which reduce penetration costs, but may lead to imitation/appropriation by local rivals.

The choice between export and FDI will be made considering internal and external aspects of the company. Exports are facilitated by technological developments and international trade regulations that contribute to reductions in transport, telecommunications, and tariffs, and to tax harmonization, and reduce the interest in diversifying production units (FDI). If there are obstacles to exports, companies may use agents or local importers to offer their products (Arm’s length). However, there are products and services that, by their nature, are difficult to supply in this way, namely when it is necessary to provide an after-sales service, adaptation of products, or when there is a strong component of brand reputation. Thus, many cases of vertical and horizontal integration resulted from the need to prevent problems arising from uncertainty, or increased costs arising from commercial mechanisms (which may result from geographic or cultural distance, differences in the legal framework or institutional dimension). In short, factors such as tariffs and quotas, discrimination against foreign companies, transport costs, distance, strong competition, high technology, and experience in foreign markets tend to favor foreign direct investment. On the contrary, exports are favored by restrictions on the possession of foreign capital and by political and exchange rate risks.

An underlying issue of the internationalization process is its genesis, since, in some cases, the internationalization process is the result of chance, although typically involves a deliberate strategy. Each internationalization strategy can be described as the association between some important internal advantages of the company (I) and external factors (E), where:

KH - Know-how, knowledge, and accumulated experience.

MF - Available financial means (internal and external).

SM - Access to Markets.

CP - Installed production capacity, infrastructures that enable production.

ME - External environment, context in which the company operates, that is likely to motivate FDI (legislation, Government support and incentives, etc.).

Crossing I and E ( Table 1), the main diagonal does not appear be of much relevance since it corresponds to a reinforcement of the advantages that already exist at the home market. The SM column represents the conquest of more attractive markets.

**Table 1.** Matrix of the determinants of FDI.

E	KH	SM	MF	CP	ME
**I**					
**KH**	KH; KH	KH; SM	KH; MF	KH; CP	KH; ME
**SM**	SM; KH	SM; SM	SM; MF	SM; CP	SM; ME
**MF**	MF; KH	MF; SM	MF; MF	MF; CP	MF; ME
**CP**	CP; KH	CP; SM	CP; MF	CP; CP	CP; ME
**ME**	ME; KH	ME; SM	ME; MF	ME; CP	ME; ME

Adapted from Valadares *et al*. (1995) with the introduction of variable ME. KH - Know-how; MF - financial means; SM - Access to Markets; CP - production capacity; ME - External environment.

The CP column means the use of installed capacity abroad associated with home production capacity, know-how, market, financing, and the national environment. In this case, the investment must target host countries with lower costs or underutilization of existing production capacity, due to industrial conversion processes. The KH column is related to access to foreign know-how to leverage the advantages acquired internally. The MF column is related to easier/cheaper financing abroad and the projection of national competences abroad to reduce financing costs. Finally, the ME column represents situations of granting incentives by foreign authorities, favorable legislation to attract investment in the host countries and/or the presence of other national firms in the host country (suppliers, customers, competitors) with which the company has strong links.

**Foreign investment objectives.** Foreign direct investment may aim at commercial expansion or at rationalizing costs using cheaper production factors. Commercial expansion may become the main objective when the capacity of the domestic market is weak or there are high transport costs, restrictions on foreign trade or restrictions imposed by consumers (nationalism, product image, uncertainty of supply or the need to product adaptation) or when firms need to follow their competitors/customers. Investments aimed at taking advantage of production factors are motivated by difficulties in accessing production factors or differences in their cost in the country of origin, rationalization of production, vertical integration, high installed capacity without corresponding demand in the domestic market, incentives or due to the advanced stage of the products’ life cycle. However, currently, these two objectives tend to be diluted, since some host countries have become important both for cost rationalization and as new markets.

## Methods

### Study design and questionnaire

Quantitative research was conducted through a survey applied to a non-probabilistic sample of managers, specifically targeting managers of Portuguese firms investing in Poland, Hungary, or the Czech Republic. The inclusion criteria were established to ensure relevance to the research objectives and focused on managers within the specified context.

The survey methodology, based on Bartels *et al.* (2009), involved the distribution of questionnaires by the first author, who also provided explanations to the respondents. The completed questionnaires were collected just before conducting subsequent interviews. To ensure internal consistency, data from the questionnaires were introduced into STATA 17.0 for processing. The selection of variables, covering motivation, determinant factors, and barriers, was informed by the theoretical framework analyzed in the previous sections.

The survey questionnaire, structured with **Likert scale questions across four groups**, aimed to explore identification, motivation, determinant factors of attraction, and barriers to investment abroad. Due to the initially extensive number of variables ( **70 items**), meeting the recommended minimum number of respondents for factor analysis was deemed impractical for the dataset. The limitations of the database in terms of sample size precluded the application of factor analysis, as recommended in the literature. The reliability of the questionnaire was evaluated using **Cronbach’s alpha, resulting in a value of 0.87**, indicating high reliability after reducing items in groups 2, 3, and 4. The detailed questionnaire is accessible in **Extended Data ( Santos, 2022c)**, with validity dimensions outlined in Table 3 **of the appendix**.

The population comprised 28 Portuguese companies investing in at least one of the three countries, with 15 investing in Poland, 10 in Hungary, and 7 in the Czech Republic. Some firms invested in multiple countries. Data from the Portuguese Investment Agency provided insights into the sectoral and spatial distribution of the known population of firms, as displayed in Table 3 in the appendix. This table indicated that manufacturing industries represented 53% of the population, reflecting the diverse investment areas. Notably, Poland captured more sectors, as elucidated through an interview with the manager of a construction company investing in all three countries. According to the manager, “In objective terms, by descending order, the most developed is the Czech Republic, followed by Hungary and Poland, but the country that most matters for us is Poland because it is the largest.”

In the sample selection process, it is essential to note that the small sample size may compromise generalizability due to its limited size and diversity. However, justifications for the sample size include the lack of a feasible secondary data source and alignment with the average range observed in comparable studies. The results, while influenced by the sample size, provide valuable insights consistent with existing research, justifying their presentation and discussion.

#### Interviews

The second research component involved exploratory semi-structured interviews with male managers at companies’ headquarters. The interviews aimed at obtaining a comprehensive understanding of motivations and difficulties related to investments in Poland, Hungary, and/or the Czech Republic. The open-ended nature of the initial question allowed for a nuanced exploration of the investment decision-making process. In the interviews, questions were posed in a way that did not influence responses, allowing for an open discussion about the investment process. The interviews facilitated a SWOT analysis of the recipient countries, contributing to a comprehensive understanding of the external environment. The data analysis process involved recording and transcribing interviews, followed by a line-by-line analysis according to Grounded Theory. This methodology aimed to identify main concepts and structure the logic underlying investment decisions. Ethics and consent procedures involved obtaining oral consent, audio recording for documentation, and approval from the ethical review board in March 2022. The decision for oral consent was made for process simplification and was deemed appropriate by the respondents.

## Results

### Characteristics of firms in the sample

The firms vary in age, experience, and strategies (see Table 2). The construction and financial sectors show a focus on international expansion, strategic risk management, and leveraging partnerships. In the electric industry, firm C emphasizes assembly and internationalization. In retail, firms D and F strategically invested in Poland, focusing on independence and product differentiation, respectively.

**Table 2.** Characteristics of respondent Companies: Industry, Age, and Strategic Focus.

Company	Sector	Age	Strategic Focus	Key partnerships	Key Markets
A	Construction	78	Resilience, International Expansion, Strategic Foresight	Local entities	Hungary, Slovakia, Czech Republic, Poland
B	Financial	37	Strategic Risk Management, Market Testing	Portuguese banks	Hungary
C	Electric	n/a	Assembly, International Expansion, Diversification	Joint venture	Czech Republic
D	Retail	38	Independence, Product Differentiation	Consortium	Poland
E	Financial	26	Integration into Common Market, Large Consumer Base	Well-established Polish bank, KBC	Poland
F	Retail	232	Product Differentiation, Cost Reduction	Consortium	Poland, Hungary

Source: Own analysis.

Company A, with 78 years of experience, is an established player in the construction industry. The company has a rich history and has demonstrated resilience through various market conditions. It strategically expanded internationally, focusing on Central Europe, especially in Hungary, Slovakia, the Czech Republic, and Poland. The decision to invest in these countries proved prescient, reflecting strategic foresight. The company competes on equal footing with German and Austrian counterparts, leveraging local entities and trained staff for a competitive edge.

Company B, a 37-year-old player in the financial sector, faced volatility in financial performance during crisis periods, highlighting the importance of strategic risk management. The company strategically invested in Hungary, emphasizing caution and meticulous market testing. The focus on reducing bad debts through automatic debits and exploring funding sources like securitization indicates a proactive risk management approach. The strategic vector involves international expansion, leveraging relationships with Portuguese banks for market entry into new regions.

Company E, a 26-year-old entrant in the financial sector, invested in Poland, recognizing its potential for integration into the common market and a large consumer base. The decision was influenced by a partnership with a well-established Polish bank and the support of KBC, a Belgian bank. The company faced strong competition in the Polish banking market and aimed to transfer successful products from Portugal, emphasizing technology transfer and synergies with the Belgian partner.

Company C operates in the electric industry, emphasizing assembly and international expansion. The company focuses on both upstream and downstream activities. It strategically diversified into the Czech Republic through a joint venture, aiming to benefit from lower production costs and navigate challenges in the domestic market. The company’s strategy involves international expansion and joint ventures to seize opportunities in Eastern European countries.

Company D with 38 years of experience, is an established player in the retail sector. The company, part of a consortium, strategically invested in Poland to tap into the growing market in Eastern Europe. The focus on direct reach to local consumers, infrastructure investment, and promoting Portuguese footwear exclusively reflects a strategic approach to gain independence from large foreign multinationals.

Company F a retail giant with 232 years of history, faced stagnation in Portugal, leading to an investment strategy in Poland and Hungary. The company strategically positioned itself as the only Portuguese textile distributor in these markets, leveraging product differentiation and cost reduction efforts. The positive evolution in sales indicates successful market entry, and the company aims to continue its growth trajectory independently.

### Interview results

**Figure 1.**
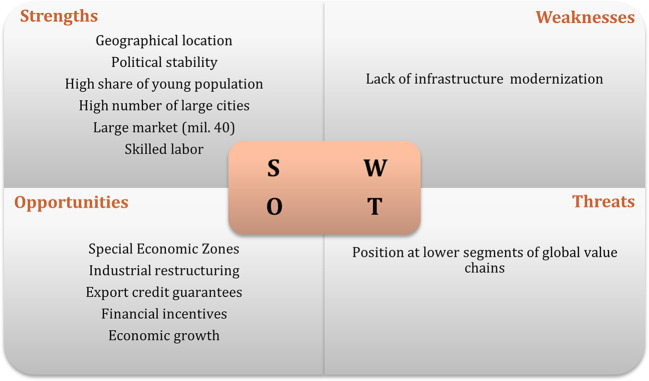
SWOT analysis for Poland.

**Figure 2.**
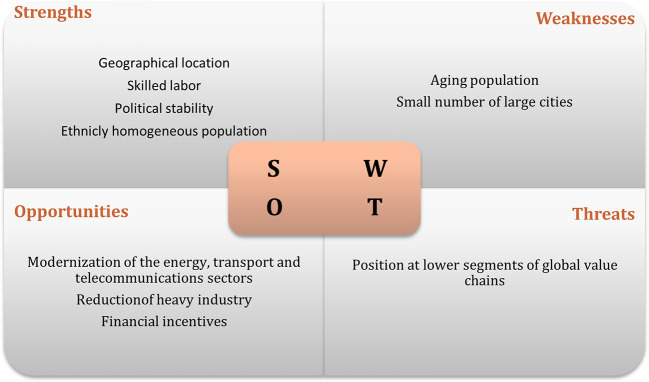
SWOT analysis for Hungary.

**Figure 3.**
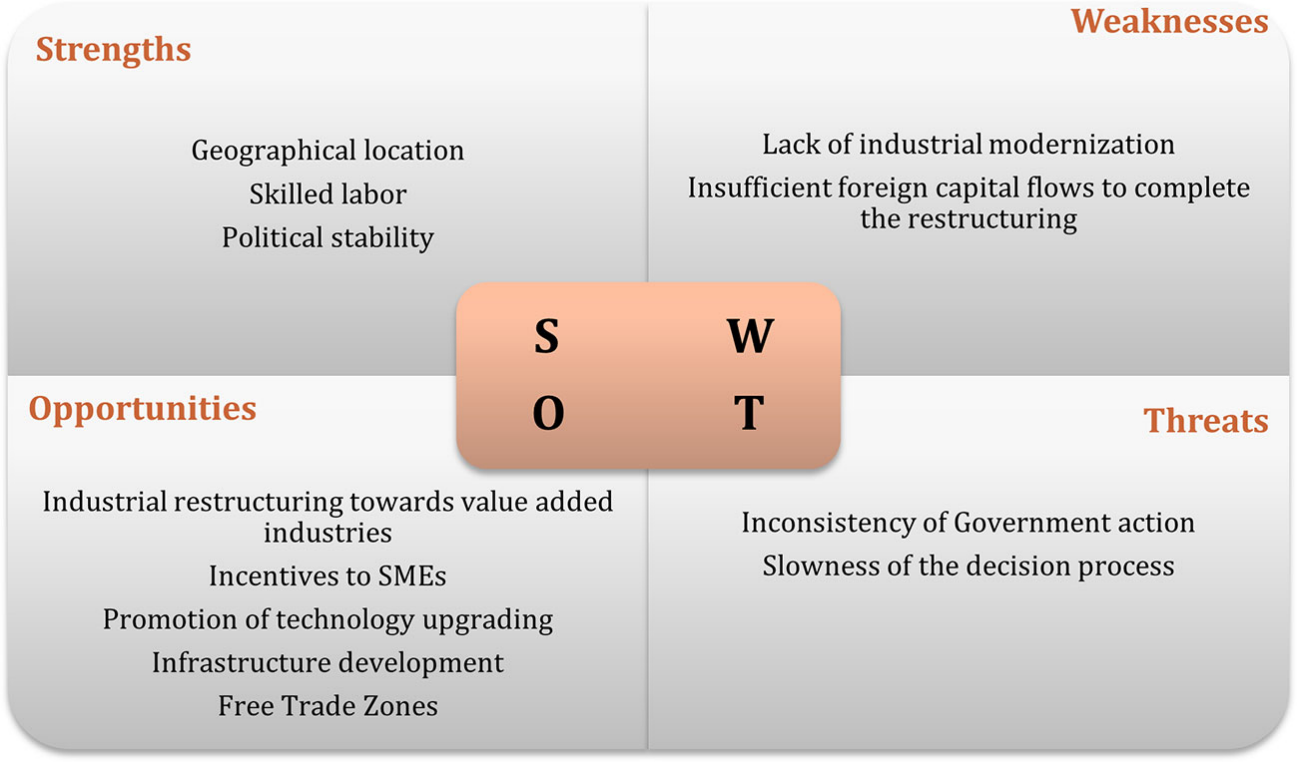
SWOT analysis for Czech Republic.

It was only possible to carry out six interviews, due to the lack of response ( Santos, 2022a). The results of interviews regarding the swot analysis of the recipient countries are displayed in Figures 1 to 3. The figures show that the three economies benefit from common strengths such as geographical location, skilled labor, and political stability.

The figures illustrate that all three economies share common strengths such as geographical location, skilled labor, and political stability. However, Poland exhibits six strengths, while Hungary and the Czech Republic show five and three strengths, respectively. In terms of threats, Poland and Hungary face similar challenges related to their position in the global value chain, while the Czech Republic faces threats associated with political processes.

Weaknesses vary among the countries, with the lack of modernization being a common weakness for Poland and the Czech Republic, while Hungary faces challenges such as an aging population and limited urban centers. Opportunities are abundant, particularly in Poland and the Czech Republic, with common opportunities including special economic zones, industrial restructuring, and technological upgrading. These findings suggest that Poland is the most attractive destination for Portuguese investment, followed by Hungary.

The sample of firms demonstrates their ability to leverage domestic skills in foreign markets, all employing active internationalization strategies. Table 5 in the appendix presents attraction factors, investment motivation, entry modes, objectives and strategies pursued, competitive advantages, threats, and challenges. Competitive advantages such as installation and anticipation over competitors are prevalent, with firms E and F exhibiting the highest number of competitive advantages. Strategies vary, with the textile industry demonstrating the greatest diversity.

Motivations for investment vary, with firm E showing a broader range of motivations including globalization and risk diversification. Attraction factors such as market size and political stability are significant, particularly for firms A and E. Threats, mainly competition, are common, with firm C facing additional challenges related to partner experience. Challenges include bureaucracy and human relations, particularly for firms C, D, and F.

Applying the information from the interviews to the Matrix of the determinants of FDI ( Table 1), the results are shown in Tables 6 to 11 (in the appendix) and summed-up in Table 12.

**Table 12.** Determinants of FDI for the sample of firms.

E	SM	CP	MF	KH	ME
**I**					
**SM**	A+B+D+E+F	A+B+E			A+B+D+E+F
**CP**	A+B+C+D+E +F	A+B+C+ E			A+B+C+D+E+F
**MF**	A+B+C+D+E +F	A+B+C+ E			A+B+C+D+E+F
**KH**	A+B+C+D+E +F	A+B+C+ E			A+B+C+D+E+F
**ME**	A+D				A+D

This analysis reveals that firms leverage internal advantages such as market position and production capacity to access foreign markets. Entry modes vary, with M&As and joint ventures being common.

Market-oriented strategies prevail, with firms capitalizing on their environment. Internal competencies such as financial means, know-how, and production capacity are ubiquitous. The interviews offer valuable insights into the motivations, challenges, and strategies of Portuguese firms investing in Central and Eastern Europe.

### Surveys’ results

It was only possible to carry out seven surveys, due to the lack of response ( Santos, 2022b). We calculated the medians of the Likert scale for each item/group. Starting with Motivation (group 2), among the main reasons for investing in those countries are marketing advantages, the importance of the foreign market in terms of turnover and profit. These findings are related to the nature of the sample of sectors and because most companies invest in Poland, the larger market of all three. Indeed, investments directed to Poland are mostly market seeking, with a view to supply the neighboring countries. Motivations such as the need to reduce the costs of supplying foreign markets, adapting products, counterattacking foreign rivals, reducing information costs, mitigating the uncertainty of the supply, eliminate transport costs, improve product quality, overcome non-tariff barriers or nationalism were not considered relevant by the respondents. This suggest that the investment was not motivated by difficulties in the market of origin or arising from exports, rather fit into a logic of supplying local markets.

**Figure 4.**
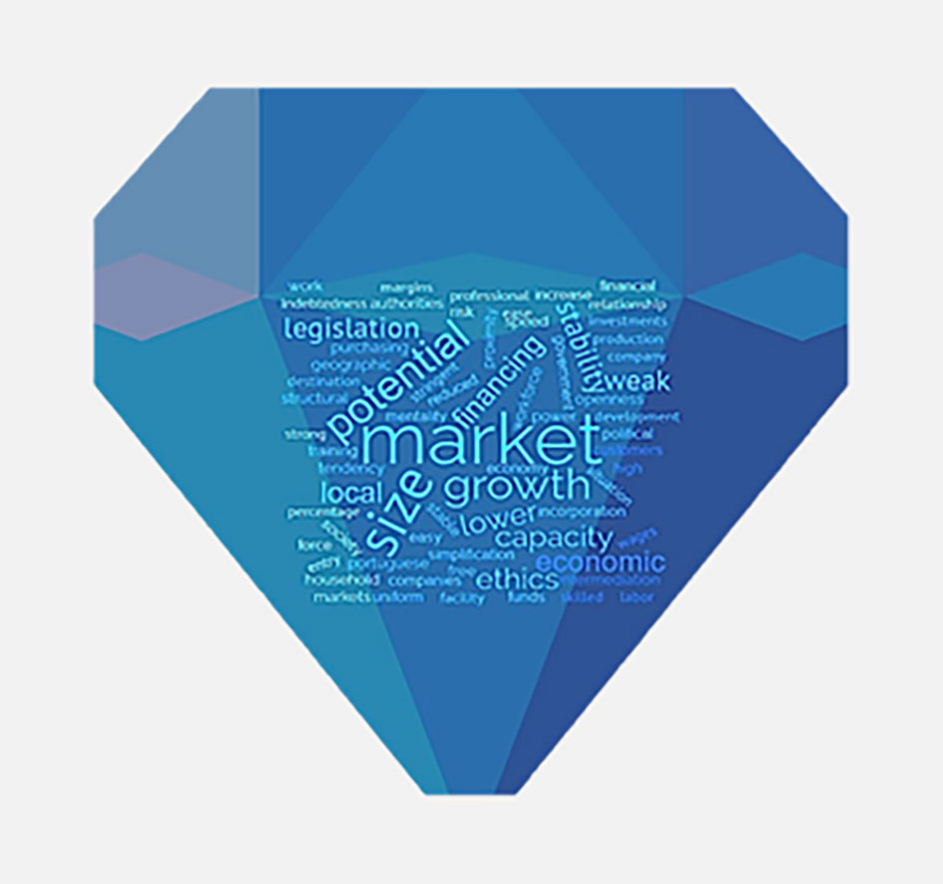
Factors of attraction of Portuguese direct investment in the Central and Eastern European Countries.

The main attraction factors are the distribution channels, the size and the growth potential of host countries’ markets, the institutional framework, and the reduced political risk ( Figure 4).

The wages in host countries were not considered relevant. Resmini (1999) comes to the same conclusion when stating that the low cost of labor is not an important objective for the FDI, given that it is oriented towards the long run when wage differentials tend to disappear.

Other authors highlighted the fact that, once the location decision has been made, in a country with low labor costs, the search for labor at the lowest possible cost may or may not be important. Natural resources are considered non-relevant for the investment, since none of the companies adopted a resource seeking strategy.

The survey’s results indicate that the role of government regarding financial incentives has been redundant for the investment decision. This is in line with the idea that investments must be profitable per se and not rely on state aid. In addition, the changing nature of incentives over time may introduce an element of mistrust among investors. Language and the existence of complex legislation were mentioned as the main challenges/difficulties that may occur in the investment process. However, culture is not considered a major challenge, which can be explained, in part, by openness of society in those countries. Real estate and financing were not considered difficulties since the main forms of market penetration were M&As and joint ventures, and Portuguese banks appear to be following their clients to these countries.

## Discussion

The empirical findings generally align with the hypotheses and underlying theories, providing a robust foundation for understanding the determinants and motivations behind Portuguese investments in Poland, Hungary, and the Czech Republic. The results contribute to a nuanced comprehension of how firms navigate challenges and leverage opportunities in their internationalization endeavors.

The survey results support H1, as the main attraction factors identified include the distribution channels, size, and growth potential of the host countries’ markets. The institutional framework and reduced political risk are also highlighted, aligning with the economic and political stability aspects emphasized in the Eclectic Paradigm (OLI Framework) and Uppsala Model.

The survey results confirm H2, as the respondents cite marketing advantages, the foreign market’s importance in terms of turnover and profit, and the need to reduce the costs of supplying foreign markets. This aligns with the Motivation for FDI Theory, suggesting that firms invest abroad to capitalize on identified business opportunities and leverage the quality-price ratio of the workforce.

The survey findings provide support for H3, as the companies exhibit a market-oriented strategy by emphasizing factors like distribution channels, market size, and growth potential. This aligns with the Resource-Based View (RBV), indicating that firms leverage their internal strengths for competitive advantage, focusing on growth and expansion.

The results confirm H4, as the competitive advantages identified by the firms include installation, anticipation over competitors, experience, product adaptation, design, quality, credibility, price, financial incentives, and fame/tradition. These align with the Competitive Advantage Theory (Porter’s Diamond Model), suggesting that anticipation of installation, experience, and product adaptation are crucial sources of competitive advantage.

The survey results validate H5, as language and the complexity of legislation are mentioned as significant challenges. This aligns with Transaction Cost Economics (TCE), indicating that challenges related to information asymmetry and contracting, such as language barriers and legislative complexity, influence the mode of entry and the decision to internalize operations.

The results from both the interviews and surveys provide support for the Eclectic Paradigm (OLI Framework) and Uppsala Model. Economic and political stability, market size, and growth potential in the CEE countries emerge as critical location factors, influencing Portuguese firms’ decisions to internalize operations. The incremental and experiential nature of internationalization is reflected in the firms’ strategies, supporting the Uppsala Model.

In particular, our results corroborate the findings of several authors. First, Market size is an important attraction factor for FDI ( Azam & Lukman 2010; Curran *et al*., 2017). Second, there is no evidence that FDI was induced by tariffs in the host countries ( Agodo, 1978; Moore, 1993). Third, human capital is less important than the market for investment decisions in developing countries ( Deyo, 1989). Fourth, political events (e.g., nationalization of foreign-owned assets) can disrupt the economies by jeopardizing past investments ( Castro, 2000). Bartels *et al*. (2009) showed that FDI in ten Sub-Saharan African countries was mostly motivated by political economy considerations, rather than by labor and production input variables. Also, Institutional factors (good government governance, economic freedom, public efficiency) play an important role in FDI decisions ( Dixit, 2011). Tomelin *et al*. (2018) and Minh (2019) conclude that institutional quality (legal structure, strong property rights, freedom to trade, and civil liberties) is important for FDI attraction. Gubik *et al*. (2020) highlighted tax optimization, geographical distance, and global production chain as major motivations for FDI in the Visegrad countries (Czech Republic, Hungary, Poland, and Slovakia). Sixth, incentives were not a relevant determinant of FDI ( UNCTAD, 1998).

By contrast, we could not find evidence to support the findings of several authors. First, contrary to Castro (2000), the incentives to foreign investors were not important instrument of investment policies. Second, we could not support the idea that the availability of raw materials and the cost and labor supply have a major impact on FDI decisions as highlighted by Dunning (1988). The same happens for the transport costs pointed out by Dunning (2015). Also, Tsai (1994) found that lower labor costs are important to attract FDI to economies. Third, we could not support the argument that culture is the core motivations for FDI, unlike Saleh *et al*. (2017). Fourth, Hirsch *et al*. (2020) found that water resources and land abundance are major determinants of FDI contrary to our results.

Unlike Kedia *et al*. (2012) we could not support the point of view that many firms start as niche providers for their rivals that are already established in the market, and as their role becomes more important in these firms’ value chain, they may aim at acquiring the ability to compete in product development abroad.

Also, unlike Leahy & Pavelin (2003) we did not find that domestic competitors have followed the leader abroad to facilitate collusive behavior in the markets in which they compete. In manufacturing and service industries, firms may follow their customers abroad to keep them ( Kim & Rhee, 2009). However, that was not the case of managers that were interviewed. Finally, the quality of the workforce is pointed as another factor of attraction of FDI ( Chansomphou & Ichihashi, 2011). Chakraborty *et al*. (2018) states that labour efficiency is more important to attracting foreign firms than Infrastructure, in India. We could not corroborate these findings.

The viability of the internationalization model requires the analysis of the main agents that compete for its implementation: consumers, competitors, foreign capital, and national and foreign authorities. Although each agent can intervene differently, depending on their objectives and constraints, some dominant behaviors can be typified: consumers and national authorities are receptive to FDI; competitors consider it a threat; and foreign authorities are faced with a dilemma of defending domestic firms/attracting FDI. In this framework, industrial policy plays an important role by contributing to the achievement of higher levels of competitiveness through the increase of manufacturing productivity ( Santos & Khan, 2019). Results suggest that investments were market-oriented aiming at expansion and profit. This requires firms’ competitiveness, which involves preserving the economic sustainability of firms ( Santos & Moreira, 2022). In this context, the vast Polish market was the one that most attracted Portuguese investors. The lower GDP per capita in Poland compared to the other two economies, suggests that the size of the potential market overcame consumer purchasing power considerations in the investment decisions. State aid, in turn, only played a supporting role in the investment, not constituting, in any case, the engine of that process. As the manager of company D said: “… no investments were made because of incentives. Many people invest because they will have support. This does not work. Investments must make sense per se. Investments should be viable or else, one can have all support in the world, and it still do not work. Off course, the support is very welcome, and sometimes can leverage these projects to succeed and consolidate faster.”

The hybrid feature of some strategies can align with the cautious attitude towards the investment (risk aversion), translated into cooperation agreements with financial institutions for funding, the market learning process, and the training of the personnel.

The anticipation of the installation over potential competitors, the experience in production and international markets, the price-quality ratio, the capacity of product adaptation and the design were considered important sources of competitive advantage that motivated the investment. The greatest difficulties during this process were language and the complexity of legislation.

*Limitations.* As mentioned above, the small number of respondents makes it difficult to use modern statistical methods of analysis such as factorial analysis. We carried out a summary of the content of interviews without using qualitative software (e.g., Maxqda, NVivo, etc.). Since the sample represents only 25% of the population, it is not representative (in terms of size and diversity of activities) which can compromise the reliability of the conclusions. Thus, we can only expect to provide hints on the outward investment process. Also, the analysis of the matrix of determinants of FDI is characterized by an excessive simplification that can be overcome through a multiple analysis, combining, in each column or row, several internal advantages/external attraction factors. Due to the importance given to the investment process in unknown foreign environments, it seems more realistic to admit that firms only decide to invest abroad when there are strong competitive advantages to offset the disadvantages or when there is more than one internal advantage and/or external attraction factor. The absence of information about the firms, such as size, age, and internationalization level, may limit the depth of insights into the specific characteristics of the studied firms. This limitation is important to consider when interpreting and generalizing the results, as a more nuanced understanding of individual firms could provide valuable context for the findings. Also, the absence of specific details about the firms hinders the ability to draw meaningful connections with prior research. Lastly, the study is constrained by the absence of econometric modeling. The reliance on a predominantly qualitative approach may limit the statistical rigor and depth of analysis. A more quantitative methodology, incorporating econometric modeling, could offer enhanced precision and robustness to the study’s findings. Future research considering a mini-panel approach could address this limitation by providing a more holistic understanding of the phenomena under investigation.

## Conclusion

Despite the business opportunities, Portuguese investment in Poland, Hungary, and the Czech Republic remains negligible, largely attributed to the geographic and cultural distance. Nevertheless, the allure of economic and political stability, coupled with the substantial market size and growth potential, serves as undeniable attraction factors for Portuguese investors. This is particularly true for Small and Medium-sized Enterprises (SMEs), which, owing to their adaptable conditions facilitating changes in activities, are the primary focus of international investment. The banking and financial sector, reflecting a broader trend towards outsourcing, especially at the expense of traditional manufacturing industries, exhibits heightened dynamism. Conversely, the decline of large heavy industries with significant investments in physical capital has prompted a shift towards diversification strategies. Companies are now directing their focus towards new products and technologies, particularly those related to information processing, telecommunications, and robotics. This strategic realignment aims to fortify competitive positions and may involve the establishment of networks by Portuguese firms, leveraging diverse production conditions in the Central and Eastern European Countries (CEECs). The accession to the European Union (EU) has facilitated these countries’ capacity to attract FDI. However, the smaller size of domestic markets, especially in the cases of Hungary and the Czech Republic, could diminish their attractiveness for market-seeking investments if more profitable alternatives exist through imports. This scenario might lead to divestments resulting from internal productive reorganization.

The qualitative and quantitative evolution of outward investment from Portugal hinges on investor motivations and the prevailing conditions offered to them. It becomes evident that Portuguese companies navigate a complex global landscape influenced by factors such as the evolution of international competition, global efforts to attract investments, and the quality of infrastructure in host countries. The originality and research value of this study lie in shedding light on the intricate dynamics shaping Portuguese investment decisions in the CEECs. Thus, it becomes imperative to address how policymakers can foster an environment conducive to increased Portuguese investment in these countries. Exploring avenues for future research can enhance the depth and applicability of the study on Portuguese outbound investment. Incorporating a more quantitative methodology, including econometric modeling, would bolster precision and robustness in examining the factors influencing investment decisions. A longitudinal study could offer valuable insights into the evolving dynamics of market conditions and opportunities over time, providing a nuanced understanding of the investment environment. Additionally, expanding the scope to include a comparative analysis of Portuguese investment in the Central and Eastern European Countries (CEECs) with other European nations would provide a broader context for identifying unique influencing factors. Investigating the role of SMEs in network development within the CEECs offers a chance to understand how these businesses establish and leverage networks, shedding light on their resilience in foreign markets. By pursuing these research directions, the academic community can contribute significantly to a more profound understanding of the complexities surrounding Portuguese outbound investment in an ever-evolving global landscape.

## Data availability

### Underlying data

Figshare: Interviews. Challenges https://doi.org/10.6084/m9.figshare.20057027.v1 ( Santos, 2022a).

This project contains the following underlying data:

• Interviews.pdf (interview transcripts)

Figshare. Dataset. https://doi.org/10.6084/m9.figshare.20433141 ( Santos, 2022b).

• Raw responses.csv (responses to survey)

### Extended data

Figshare: Questionnaire. https://doi.org/10.6084/m9.figshare.20358987.v1 ( Santos, 2022c).

• Questionnaire.pdf

Figshare: Tables 3 to 12. https://doi.org/10.6084/m9.figshare.25195733.v2 ( Santos, 2024).

• Appendix F1000. docx

Data are available under the terms of the Creative Commons Attribution 4.0 International license (CC-BY 4.0).
