# Supplementary material for: Outward investment of Portuguese small and medium enterprises in the Central and Eastern European countries: motivations and challenges
Source: F1000Res. 2024 May 31;11:1339. Originally published 2022 Nov 17. [Version 3] doi: 10.12688/f1000research.122697.3 (PMC11222775; doi:10.12688/f1000research.122697.3)
Supplement: Peer review history

# Reviewer report: Reviewer response for version 3

**DOI:** 10.5256/f1000research.166923.r285381

**Published:** 2024-07-03

**Authors:** Petr Hlavacek

**Affiliations:** 1 Jan Evangelista Purkyne University, Ústí nad Labem, Czech Republic

I have reviewed your revised manuscript and I inform you that I accept your response. The amendments have addressed the identified weaknesses, and the revisions enhance the clarity of the study.

 I recommend this article for approval.

Is the work clearly and accurately presented and does it cite the current literature?

Yes

If applicable, is the statistical analysis and its interpretation appropriate?

Partly

Are all the source data underlying the results available to ensure full reproducibility?

Yes

Is the study design appropriate and is the work technically sound?

Yes

Are the conclusions drawn adequately supported by the results?

Yes

Are sufficient details of methods and analysis provided to allow replication by others?

Partly

Reviewer Expertise:

spatial economics, regional development

I confirm that I have read this submission and believe that I have an appropriate level of expertise to confirm that it is of an acceptable scientific standard.

# Reviewer report: Reviewer response for version 2

**DOI:** 10.5256/f1000research.162460.r266088

**Published:** 2024-05-09

**Authors:** Petr Hlavacek

**Affiliations:** 1 Jan Evangelista Purkyne University, Ústí nad Labem, Czech Republic

The article examines the motivations and challenges of Portuguese small and of Portuguese medium-sized enterprises (SMEs) when investing in Central and Eastern Europe, particularly in Poland, Hungary and the Czech Republic. The study is based on interviews among Portuguese business managers. enterprises. The strength of the paper is the integration of theoretical approaches in the context of foreign direct investment. The weaknesses of the paper are the small sample of respondents and the  responses, I also recommend adding a table with the characteristics of the respondents subjects. The Financial Sector chapter does not address the topic at all, it does not belong in the article. The chapter Retail (textiles and footwear) is also problematic. I recommend revised both chapters.

Is the work clearly and accurately presented and does it cite the current literature?

Yes

If applicable, is the statistical analysis and its interpretation appropriate?

Partly

Are all the source data underlying the results available to ensure full reproducibility?

Yes

Is the study design appropriate and is the work technically sound?

Yes

Are the conclusions drawn adequately supported by the results?

Yes

Are sufficient details of methods and analysis provided to allow replication by others?

Partly

Reviewer Expertise:

spatial economics, regional development

I confirm that I have read this submission and believe that I have an appropriate level of expertise to confirm that it is of an acceptable scientific standard, however I have significant reservations, as outlined above.

# Reviewer report: Reviewer response for version 2

**DOI:** 10.5256/f1000research.162460.r248363

**Published:** 2024-03-06

**Authors:** Viorela Ligia Vaidean, Stavila Daria

**Affiliations:** 1 Faculty of Economics and Business Administration, Babeș-Bolyai University, Cluj-Napoca, Romania; 2 Statistics, Babeş-Bolyai University, Cluj-Napoca, Cluj, Romania

Dear authors,

 I am pleased of your work, congratulations!

 Best wishes for the future!

Is the work clearly and accurately presented and does it cite the current literature?

Partly

If applicable, is the statistical analysis and its interpretation appropriate?

Partly

Are all the source data underlying the results available to ensure full reproducibility?

Yes

Is the study design appropriate and is the work technically sound?

Partly

Are the conclusions drawn adequately supported by the results?

Yes

Are sufficient details of methods and analysis provided to allow replication by others?

Yes

Reviewer Expertise:

Corporate Finance, Health Economics, Public Policies

We confirm that we have read this submission and believe that we have an appropriate level of expertise to confirm that it is of an acceptable scientific standard.

# Reviewer report: Reviewer response for version 1

**DOI:** 10.5256/f1000research.134720.r174811

**Published:** 2023-08-01

**Authors:** Viorela Ligia Vaidean, Stavila Daria

**Affiliations:** 1 Faculty of Economics and Business Administration, Babeș-Bolyai University, Cluj-Napoca, Romania; 2 Statistics, Babeş-Bolyai University, Cluj-Napoca, Cluj, Romania

The research paper titled „Outward investment of Portuguese small and medium enterprises in the Central and Eastern European countries: motivation and challenges” (F1000Research 2022, 11: 1339 Last updated 29 MAY 2023) is an extremely interesting paper which investigates the determinant factors of Portuguese direct investments (PDI) in some Central and Eastern European countries.  

 We have closely read the entire paper and have a few critical comments that we suggest to the authors:

 1. Introduction (with Literature review). The following studies need to be read and cited, as they are rather recent and related to this topic:

- https://doi.org/10.1016/j.joitmc.2023.100021
- https://doi.org/10.1016/j.ecosys.2023.101095
- https://doi.org/10.1016/j.jmacro.2012.01.010

2. Methods. Results. In our opinion, although the flow of ideas and arguments are extremely well-organised, the paper’s length might be a little overwhelming for the reader. We recommend a condensed version of these subchapters, by shortening them with one or two pages. Another variant would be to move some tables (E.g. Tables 6 to 11, keeping Table 12) into an Appendix.

 The main questionable aspect over here is the lack of econometric modelling of data. We were somehow expecting a quantitative approach from the title of your manuscript as many such papers include multivariate data analysis techniques applied to cross-sectional/panel data. Could you repeat the survey in order to structure a mini-panel approach, in the future?

 3. Surveys’ Results. Discussion. We particularly appreciate the interpretations provided by the authors.

 4. In the Conclusion the authors need to emphasize the international dimension of their findings, the originality and value of their research a little bit more. We think they should develop the policy implications of their paper and the authors’ future research directions.

**Some other comments:**

 1. Emphasize how the authors deal with the limits of this paper more.

 2. The Figshare dataset and questionnaire is rather simple. Could you extend the research towards including more respondents? Even you admit that your sample consists of “only 25% of the population, it is not representative (in terms of size and diversity of activities) which can compromise the reliability of the conclusions”

 These suggestions will improve the quality of this manuscript and they will make the paper more suitable for being published, due to its interesting results and originality.

 This review has been performed by,

 Associate Professor Viorela-Ligia Văidean, PhD

 Master degree student Daria Stăvilă

 Faculty of Economics and Business Administration

 “Babeș-Bolyai” University Cluj-Napoca, Romania

Is the work clearly and accurately presented and does it cite the current literature?

Partly

If applicable, is the statistical analysis and its interpretation appropriate?

Partly

Are all the source data underlying the results available to ensure full reproducibility?

Yes

Is the study design appropriate and is the work technically sound?

Partly

Are the conclusions drawn adequately supported by the results?

Yes

Are sufficient details of methods and analysis provided to allow replication by others?

Yes

Reviewer Expertise:

Corporate Finance, Health Economics, Public Policies

We confirm that we have read this submission and believe that we have an appropriate level of expertise to confirm that it is of an acceptable scientific standard, however we have significant reservations, as outlined above.

# Reviewer report: Reviewer response for version 1

**DOI:** 10.5256/f1000research.134720.r172426

**Published:** 2023-08-01

**Authors:** Canan Yildirim

**Affiliations:** 1 Finance and Accounting, Rennes School of Business, Rennes, France

The paper aims at finding out the determinants of outward investment of Portuguese Small and Medium-sized Enterprises (SMEs) and employs multiple case studies.

 While the subject is interesting and has important managerial and policy implications, the study suffers from several aspects.

 Most importantly, there is no theoretical framework to guide the development of hypotheses. There is no discussion of the specifics of the SMEs and the Portuguese context either. The study refers to some previous papers to develop a list of potential factors without any deeper discussions about the theory(ies) supporting these arguments. What is the research question? Why is it relevant for the SMEs in Portugal?

 The methodological approach is not carefully justified and the explanation of the actual application of the techniques is confusing at times. There are expressions such as " *our database cannot meet this criterian*", " *it was only possible to carry out six interviews, due to lack of response*". 

 The case study covers firms from diverse set of industries. Finance industry, for instance, a specific case (information intensive, regulation sensitive service industry). One would expect different drivers and mode of entry in this sector compared to others.

 There is a lack of background or additional information about these firms (size, age, overall internationalization level and experience etc.). Hence the findings seem to be very generic. I am not sure if the study's findings can be compared and contrasted with the existing literature. 

 The study might benefit from professional copy editing.

Is the work clearly and accurately presented and does it cite the current literature?

No

If applicable, is the statistical analysis and its interpretation appropriate?

Partly

Are all the source data underlying the results available to ensure full reproducibility?

Partly

Is the study design appropriate and is the work technically sound?

Partly

Are the conclusions drawn adequately supported by the results?

No

Are sufficient details of methods and analysis provided to allow replication by others?

Partly

Reviewer Expertise:

Banking, Cross-border M&As, Firm internationalization.

I confirm that I have read this submission and believe that I have an appropriate level of expertise to state that I do not consider it to be of an acceptable scientific standard, for reasons outlined above.
